# Autophagy induced by Schwann cell-derived exosomes promotes recovery after spinal cord injury in rats

**DOI:** 10.1007/s10529-021-03198-8

**Published:** 2021-11-05

**Authors:** Dayu Pan, Shibo Zhu, Weixin Zhang, Zhijian Wei, Fuhan Yang, Zhenglong Guo, Guangzhi Ning, Shiqing Feng

**Affiliations:** 1 Department of Orthopedics, Tianjin Medical University General Hospital, Heping District, Tianjin, 300052 China; 2 International Science and Technology Cooperation Base of Spinal Cord Injury, Tianjin Key Laboratory of Spine and Spinal Cord Injury, Department of Orthopedics, Tianjin Medical University General Hospital, Tianjin, China; 3 Zhejiang Chinese Medicine University, 548 Binwen Road, Hangzhou, 310053 China; 4 Department of Urology, Shanghai Tenth People’s Hospital, Tongji University, Shanghai, China; 5 Henan Provincial People’s Hospital, People’s Hospital of Zhengzhou University, People’s Hospital of Henan University, Henan, China; 6 Department of Orthopedic Surgery, Tianjin Medical University General Hospital, Tianjin, 300052 China

**Keywords:** Schwann cell, Exosome, Spinal cord injury, Autophagy, EGFR/Akt/mTOR signaling pathway

## Abstract

Spinal cord injury (SCI) is catastrophic to humans and society. However, there is currently no effective treatment for SCI. Autophagy is known to serve critical roles in both the physiological and pathological processes of the body, but its facilitatory and/or deleterious effects in SCI are yet to be completely elucidated. This study aimed to use primary Schwann cell-derived exosomes (SCDEs) to treat rats after SCI. In the present study, SCDEs were purified and their efficacy in ameliorating the components of SCI was examined. Using both in vivo and in vitro experiments, it was demonstrated that SCDEs increased autophagy and decreased apoptosis after SCI, which promoted axonal protection and the recovery of motor function. Furthermore, it was discovered that an increased number of SCDEs resulted in a decreased expression level of EGFR, which subsequently inhibited the Akt/mTOR signaling pathway, which upregulated the level of autophagy to ultimately induce microtubule acetylation and polymerization. Collectively, the present study identified that SCDEs could induce axonal protection after SCI by increasing autophagy and decreasing apoptosis, and it was suggested that this may involve the EGFR/Akt/mTOR signaling pathway.

**Supplementary Information:**

The online version contains supplementary material available at 10.1007/s10529-021-03198-8.

## Introduction

Spinal cord injury (SCI) causes severe damage to axons and results in the death of neurons, which leads to the permanent loss of motor and/or sensory function. Globally, a total of ~ 2.5 million individuals suffer from traumatic SCI, with > 130,000 new cases reported each year in the world (Holmes 2017; McDonald and Sadowsky 2002; Thuret et al. 2006). However, there is currently no effective treatment for SCI (Mothe and Tator 2012).

Exosomes are small endosome-derived vesicles (30–150 nm) that contain complex RNAs and proteins, but have more recently been referred to as disc-shaped vesicles having a diameter of 40–100 nm (Thery et al. 2002). Moreover, exosomes represent extracellular cell-derived phospholipid nanocarriers that act as signaling bodies and can deliver biologically active molecules to specific recipient cells for intercellular communication (Yang et al. 2019). It has been reported that exosomes can cross the blood–brain barrier, can enable multiple intravenous dosing without any side effects and can participate in the regulation of inflammation to promote nerve and motor functional recovery (Lopez-Verrilli et al. 2013). In the peripheral nervous system, Schwann cells promote the dedifferentiation and proliferation of axons after injury, as well as remove myelin and axon fragments. Moreover, their regenerative capacity has been applied to repair damage within the central nervous system (Webber and Zochodne 2010). Therefore, the present study further investigated the potential of Schwann cell-derived exosomes (SCDEs) in the recovery after SCI.

Autophagy is a metabolic process in which a class of intracellular substances is degraded, and autophagosomes have been observed in the nervous system (Chen et al. 2017; He et al. 2016; Yang et al. 2013). Previous studies have reported that crush injury of the optic nerve leads to the rapid entry of calcium into neurons, which subsequently induces autophagy and ultimately leads to axonal degeneration (Koch et al. 2010). Moreover, multiple studies have shown that exosomes are closely associated with autophagy. As such, the purpose of the present study was to investigate the role of SCDEs in axonal protection following SCI, as well as identify its possible mechanisms. The present findings may contribute to the development of novel therapeutic strategies for treating clinical SCI.

## Materials and methods

### Animals and injections

Female Wistar rats (weight, 240 ± 10 g; weight range, 230–250 g; n = 120) were obtained from the Laboratory Animal Center of the Chinese People’s Liberation Army General Hospital (Beijing, China). These rats were divided into the following groups (n = 6 rats/group): Sham group, without surgery and SCDEs injection; control group, injected with PBS; and SCDEs group, injected with SCDEs. The rats were housed at a temperature of 25 ± 1 °C with a 12 h light/dark cycle, and access to food and water was ad libitum. All animals were handled according to the recommendations of the National Institute of Health Guidelines for the Care and Use of Laboratory Animals. The experiments were approved by the Animal Ethical and Welfare Committee (approval no. IRM-DWLL-2017021). In total, 250 μL exosomes (0.1 μg/μL) and an equal volume of Dulbecco's Phosphate Buffered Saline (DPBS) were injected into the tail veins of the rats three times a week for 4 weeks since the induction day of SCI.

### SCI

The rats were weighed and deeply anesthetized with an intraperitoneal injection of 400 mg/kg 4% chloral hydrate. After a 1-cm skin incision was made at the T10 position of the back, the muscles of the localized area were bluntly separated to expose the T10 lamina. Subsequently, a dorsal laminectomy of the T10 vertebra was performed to expose the spinal cord. A striking rod was placed directly above the exposed spinal cord, and a 10-g node was freely dropped from a height of 2.5 cm, resulting in a model of spinal cord contusion. Finally, the muscle and skin were sutured layer by layer after all bleeding was stopped. Sodium cefuroxime was used for 3 days after surgery to prevent wound infection, and the rats were provided with manual assistance in emptying their bladders twice a day. The humane endpoints included autotomy, pain or distress, weight loss of > 20%, > 70% reduction in food intake, lethargy or apathy and severe wound scratching.

### Behavioral testing of locomotor function

The Basso, Beatlie and Bresnahan (BBB) functional score was used to quantify locomotor function (Basso et al. 1995). The test was performed before the spinal cord surgery and weekly thereafter at the same time of day and were graded by the three blinded observers. Functional restoration was assessed as per BBB locomotor scores, as previously described (Basso et al. 1995). The BBB test score evaluated hindlimb locomotor function on a scale from 0 to 21. Scoring was based on spontaneous hindlimb movement during a 5-min observation period in the open field after the animals already habituated to the arena. The animals (n = 6) were placed on a circular platform with a diameter of 2 m, and the walking and limb activities of the hind limbs were observed.

### Cell culture

The PC12 cell line was generously provided by Professor Haifang Yin from Research Centre of Basic Medical Science of Tianjin Medical University (Tianjin, China). Cells were cultured in DMEM containing 10% bovine serum, 5% horse serum, 1% l-glutamine and 1% penicillin/streptomycin. All cells were cultured at 37 °C with 5% CO_2_. For the construction of an oxidative stress model that emulates SCI in vitro, PC12 cells were incubated for 24 h in fresh medium containing 200 μM H_2_O_2_. Then, 3 mL serum-free medium containing 50 μg/L of nerve growth factor was used to induce the differentiation of PC12 cells for 5 days.

Primary Schwann cells were extracted from the sciatic nerves of adult Wistar rats and cultured in DMEM with 10% FBS and 1% penicillin /streptomycin at 37 °C with 5% CO_2_. The medium condition was changed when the cells grew up to 80% area of the culture dish. For exosomes collection, Schwann cells were cultured in DMEM supplemented with 10% exosome-free FBS and 1% penicillin/streptomycin at 37 °C with 5% CO_2_ which was obtained by centrifuging FBS at 100,000×*g* for 16 h at 4 °C. The isolated Schwann cells were characterized via co-staining of glial fibrillary acidic protein and S100 (Fig. S1A, B).

### Preparation and purification of SCDEs

The resulting culture medium of Schwann cells was harvested to obtain exosomes via multiple ultracentrifugation steps. The first centrifugation was conducted at 1000×*g* for 10 min, while the second centrifugation was performed at 10,000×*g* (Beckman Optimal-100 XP, Beckman Coulter, Germany) for 30 min. Subsequently, the supernatant was collected for the third time and ultracentrifuged at 100,000×*g* for 1 h. After washing the SCDEs with PBS, the SCDEs were obtained after a final ultracentrifugation for 1 h at 100,000×*g* and been resuspended in sterile PBS for the following experiments. The total protein concentration of exosomes was quantified using a Bradford assay (Sangon Biotech Co., Ltd.).

### Characterization of SCDEs

The size distribution of exosomes was measured using a nanoparticle tracking and zeta potential distribution analyzer (Paricle Metrix-PMX, Germany) and the morphology of the exosomes was visualized using a high-resolution transmission electron microscope (TEM, Hitachi HT7700, Tokyo, Japan). Briefly, the re-suspended exosomes were mixed with an equal volume of 4% paraformaldehyde (PFA) and adsorbed onto a glow-discharged, carbon-coated formvar film, which was attached to a metal specimen grid. Excess solution was blotted off and the grid was immersed with a small drop (50 μL) of 1% glutaraldehyde for 5 min followed by washing with 100 μL still water 2 min each time for 8 times. Subsequently, the grid was transferred to 50 μL uranyl-oxalate solution (pH 7.0) for 5 min and then 50 μL methyl cellulose-uranyl acetate (100 μL 4% uranyl acetate and 900 μL 2% methyl cellulose) for 10 min on ice. The excess solution was blotted off and the sample was dried and examined in the TEM.

### Protein extraction and western blotting

SCDEs and cell pellets were lysed with RIPA buffer, and the protein sample was then boiled and denatured in 5× SDS loading buffer for 15 min. Then, 30 µg total protein was loaded per well. Electrophoresis was performed using a 10% acrylamide gel, and proteins were transferred to a PVDF membrane on ice for 1 h. The membrane was blocked with 5% BSA at room temperature for 1 h after transfer. Subsequently, the primary antibody was incubated with the membrane overnight at 4 °C (Table SI). CD9, CD31 and Alix were picked to indicate the exosome presence (Thery et al., 2018). The following day the membrane was incubated with secondary antibody (1:2000) for 1 h at room temperature. Finally, the ECL immunoblot analysis system (EMD Millipore) was applied to visualize the bands.

### Immunofluorescent staining

In total, five rats per group and the 20th–25th slices were used for immunofluorescent staining. After anesthetizing the rats with 400 mg/kg 4% chloral hydrate and exposing the heart, a small hole was cut in the left ventricle and a needle was inserted into the aorta, then the right atrium was cut to allow flow. Perfusion with 200 mL cold saline was followed by fixing with 300 mL cold 4% paraformaldehyde at weeks 1, 2 and 4 post-SCI. The removed spinal cord tissue was 1-cm above and below the center of the lesion. Subsequently, the fixed spinal cord was dehydrated overnight in 30% sucrose, embedded and sliced into frozen sections. Frozen sections (thickness, 10 μm) were blocked with 5% donkey serum at room temperature for 1 h. Then, sections were incubated with the corresponding primary antibodies at 4 °C overnight (Table SI), followed by incubation with the corresponding secondary antibodies (conjugated to Alexa Fluor 488 or 594; Invitrogen; Thermo Fisher Scientific, Inc.) for 1 h at room temperature. Finally, the sections were observed under a LEICA fluorescent microscope.

### H&E staining

H&E staining was used to assess contusion areas and demyelination in different groups. After the rats were perfused, the spinal cord was removed at 1-cm above and below the center of the lesion and was fixed with 4% paraformaldehyde overnight, after which it was immersed in PBS. Finally, the spinal cord sample was embedded in paraffin and cut into 5-μm thick sections for H&E staining. In total, five rats per group and the 20th–25th slices were used for H&E staining.

#### Luxol fast blue (LFB) staining

LFB staining was used to stain myelin and myelinated axons. In total, five rats per group and the 20th–25th slices were used for LFB staining. The sections were added into a 0.1% LFB solution and incubated in an oven at 65 °C overnight. The following day, the sections were separated with a 0.05% aqueous lithium carbonate solution for 30 s. Subsequently, the sections were counterstained with a 0.25% tar-purple solution and a few drops of glacial acetic-acid dye solution for 10 min.

#### TUNEL staining

Sections in the center of the injury site were treated according to the manufacturer’s instructions of the DeadEnd™ Fluorometric TUNEL system (cat. no. G3250; Promega Corporation). Quantification was performed by counting the number of TUNEL-positive cells in the whole injury site on five serial sections for each rat, which was performed in duplicate.

#### Flow cytometry

The cells were incubated with 5 μL of Annexin V and 5 μL of propidium iodide (PI) for 15 min at room temperature in dark, according to the manufacturer’s instruction (BD Biosciences, SanJose, CA), and then subjected to flow cytometry to measure the apoptosis rate (%). Primary PC12 cells (1 × 10^6^) at passage 30 were suspended in DPBS and incubated with antibodies at a dilution of 1:500 for 1 h at 4 °C; isotype-matched antibodies served as negative controls. In total, three specimens were tested. Fluorescent compensation was performed using labelled controls and all measures were performed using Flow-Jo (v.7.6; Tree Star, Inc.).

#### Statistical analysis

H&E staining and LFB staining images were converted to black and white, and the area of cavitation was measured in different tissue compartments using ImageJ (v.1.51; National Institutes of Health) software. Fluorescence intensities are expressed as arbitrary units-standard errors of the mean. The cavity size for each slide was recorded using ImageJ software, and eventually, the mean cavity size for each group was measured. The cavity size was presented as percent of the total area of the section using the following formula: Cavity size (%) = [Cavity size (µm)/Total area of the section (µm)] × 100.

All data are presented as the mean ± SEM. P < 0.05 was considered to indicate a statistically significant difference. Statistical analysis was performed using GraphPad Prism 5 software (GraphPad Software, Inc.) and pairwise comparisons were performed using Student’s *t* tests. Two-way ANOVAs with Tukey’s post-hoc tests were used to determine significant differences in behaviors that included repeated measures.

## Results

### Characterizations of SCDEs

**Fig. 1:**
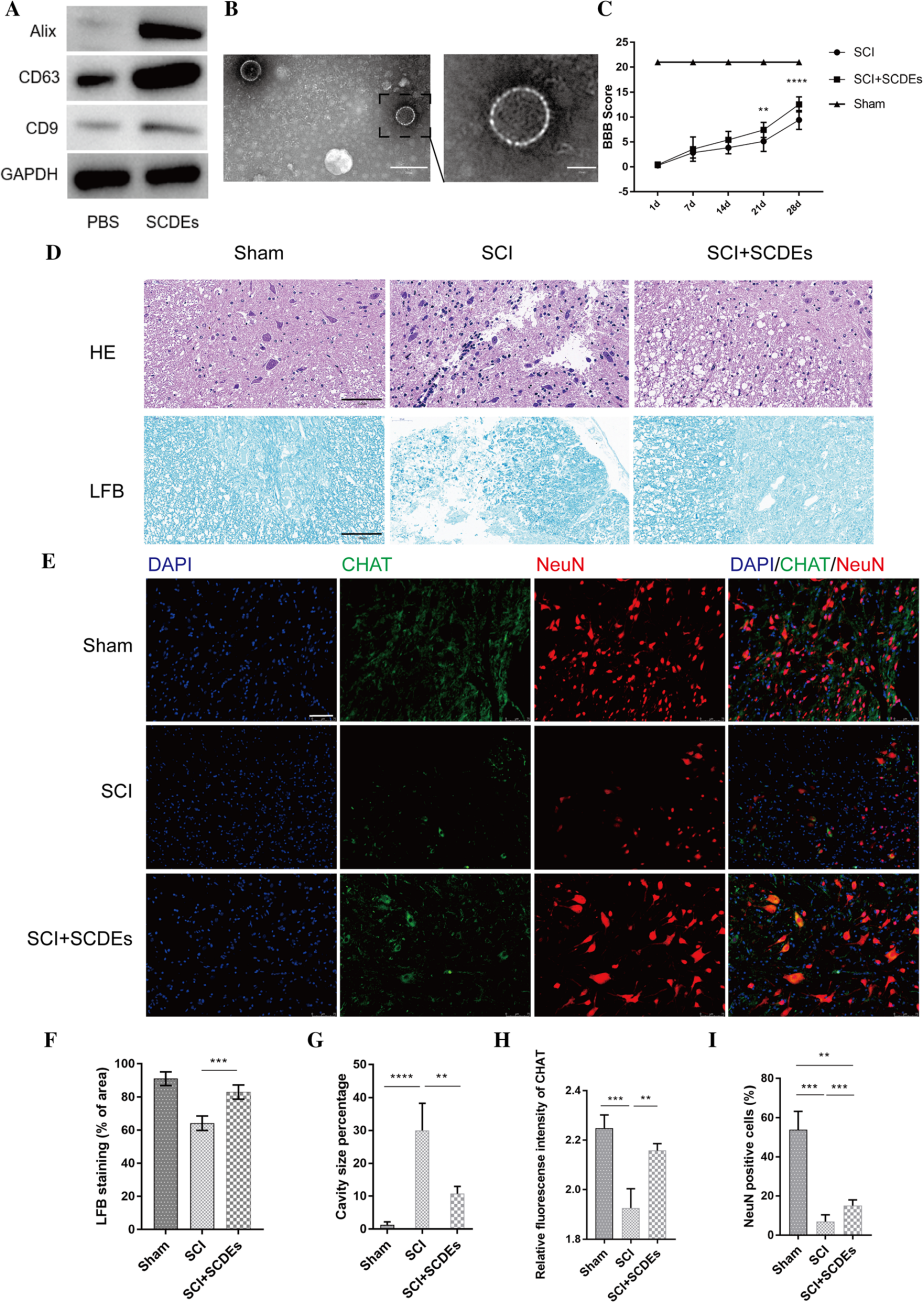
Characterizations of SCDEs, which can improve spinal cord repair after SCI in vivo. **A** Western blot analysis of the expression levels of CD9, CD63 and Alix in exosomes extracted from Schwann cells. **B** Image of exosomes under transmission electron microscopy. Scale bars denote the following: Left side, 200 μm; right side, 50 μm. **C** BBB scores are indicative of locomotor function after SCI in rats. Data are presented as the mean ± SEM; two-way ANOVA with Tukey’s post-hoc test, n = 6. *P < 0.05, **P < 0.01. **D** H&E and luxol fast blue staining of spinal cord sections at 4 weeks after surgery. Scale bar, 100 μm. **E** These cells were stained with NeuN, ChAT and DAPI. Scale bar, 75 μm. **F**, **G** Quantification of LFB staining (% of area) and cavity size percentage. **H**, **I** Quantification of the NeuN and ChAT area. Data are presented as the mean ± SEM, one-way ANOVA followed by a Bonferroni correction, n = 5. **P < 0.01, ***P < 0.001, ****P < 0.0001. *SCDEs* Schwann cell-derived exosomes,*SCI* spinal cord injury, *Alix* ALG-2 interacting protein X, *ChAT* choline acetyltransferase, *BBB* Basso, Beatlie and Bresnahan, *NeuN* neuronal nuclei

Western blotting was used to characterize the exosomes, which were found to express three exosomal markers: CD9, CD63 and ALG-2 interacting protein X (Fig. 1A). Furthermore, the size of SCDEs ranged from 40 to 100 nm, as determined via TEM, which provided additional evidence confirming that SCDEs had been isolated (Fig. 1B).

### SCDEs improve recovery of motor function after SCI in vivo

For the in vivo experiments, the recovery of rats was assessed after SCI in rats that were treated with or without SCDEs at 1, 7, 14, 21 and 28 days. There were statistically significant differences between the BBB scores of SCDEs-treated rats and sham-treated rats from day 21 (Fig. 1C), such that the BBB scores of SCDEs-treated SCI rats were comparatively improved (P < 0.05; n = 6). This finding demonstrates that SCDEs improved recovery of motor function following SCI.

### SCDEs improve spinal cord repair after SCI in vivo

Studies have reported that promoting axonal remyelination is an important part of improving spinal nerve function following SCI. H&E and LFB staining used for the evaluation of myelin content within the spinal cord revealed a noticeable rise in the percentage of myelinated areas and a significant decrease in the area of cavitation after SCDE administration in SCI rats, compared with these parameters in sham-treated SCI rats (Fig. 1D, F, G).

To investigate whether SCDEs affect the protection of injured neurological function, the expression levels of both the mature neural marker, neuronal nuclei (NeuN), and choline acetyltransferase (ChAT), which synthesizes the neurotransmitter acetylcholine in spinal motor neurons, were measured at the injury site in rats. Immunofluorescent results identified that rats treated with SCDEs expressed higher levels of both NeuN and ChAT at the injury site compared with those in the PBS-treated group, suggesting that SCDEs promoted the protection of injured axons (Fig. 1E, H, I).

### SCDE treatment after SCI inhibits apoptosis and increases autophagy in vivo

**Fig. 2:**
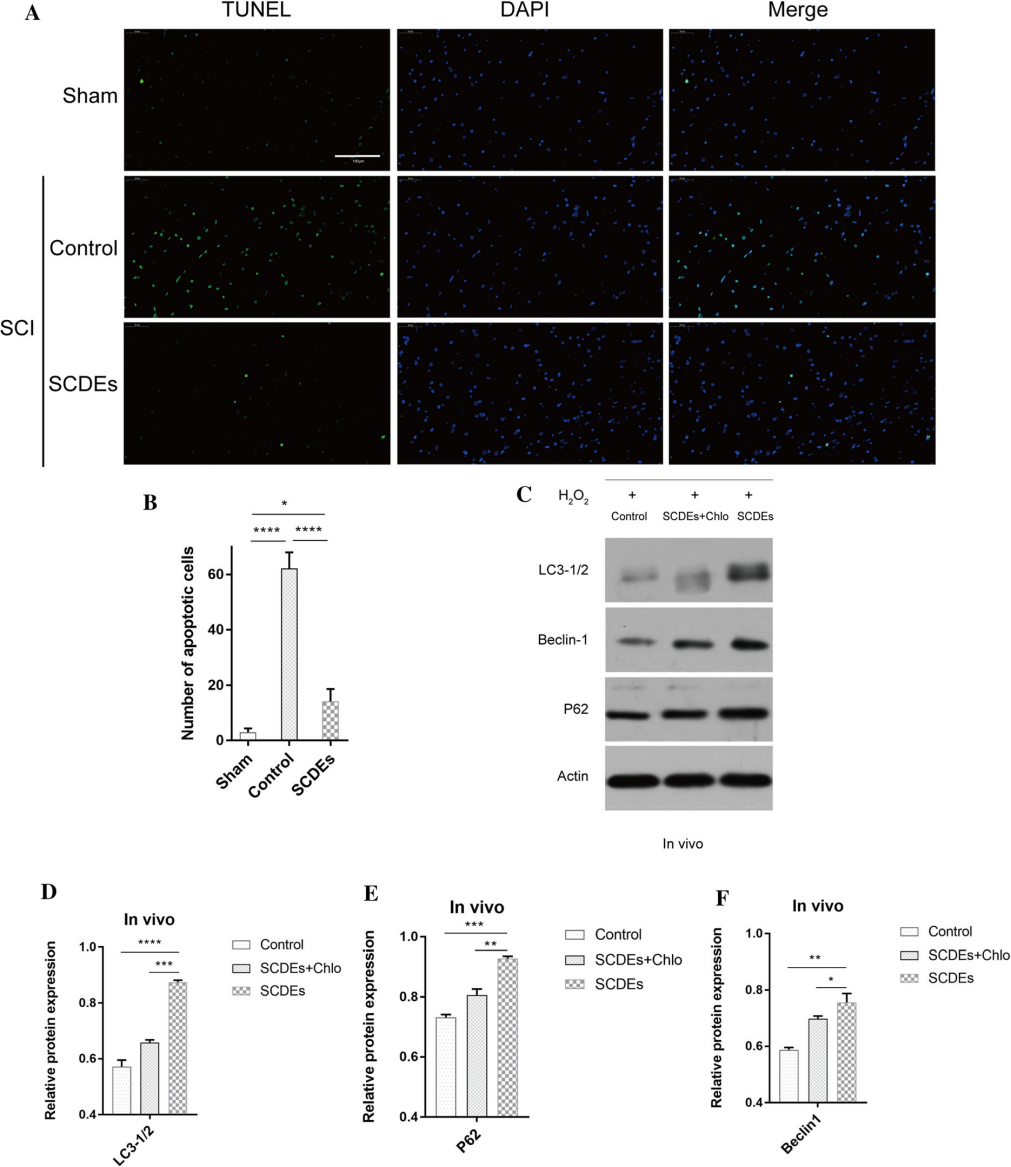
SCDEs inhibit apoptosis of injured neurons via autophagy in vivo. **A** These cells were stained with TUNEL in vivo. Scale bar, 100 μm. **B** Quantification of the number of apoptotic cells in different groups. **C** Western blotting results indicated that the SCDE-treatment group expressed higher protein levels of autophagic markers in vivo compared with those of other groups. **D**–**F** Quantification of LC3-1/2, Beclin-1 and P62 expression levels Data are presented as the mean ± SEM, two-way ANOVA with Tukey’s post-hoc test, n = 4. *P < 0.05, **P < 0.01, ***P < 0.001, ****P < 0.0001. *Chlo* chloroquine, *SCDEs* Schwann cell-derived exosomes

Next, apoptosis was measured via TUNEL assays. There was a significant increase in the number of apoptotic cells in the SCI group compared with that in the sham group (P < 0.0001), while there was a significant decrease in the number of apoptotic cells in the SCDEs-treated SCI group compared with that in the untreated SCI group (P < 0.01). These results suggested that SCDEs minimized apoptosis following SCI in rats (Fig. 2A and B).

Western blotting was used to detect the expression levels of LC3-1/2, Beclin-1 and P62, which are protein biomarkers of autophagosomes in the damaged spinal cord. The results demonstrated that the damaged spinal cord expressed higher levels of LC3-1/2, Beclin-1 and P62 in the SCDEs-treatment SCI group compared with those in the non-treated SCI group. Additionally, SCDEs-induced increases in LC3-1/2, Beclin-1 and P62 were prevented when autophagy was blocked using chloroquine (Fig. 2C–F). Therefore, these findings indicated that SCDEs may act on injured axons via autophagy.

### SCDEs inhibit apoptosis of injured neurons via autophagy in vitro

**Fig. 3:**
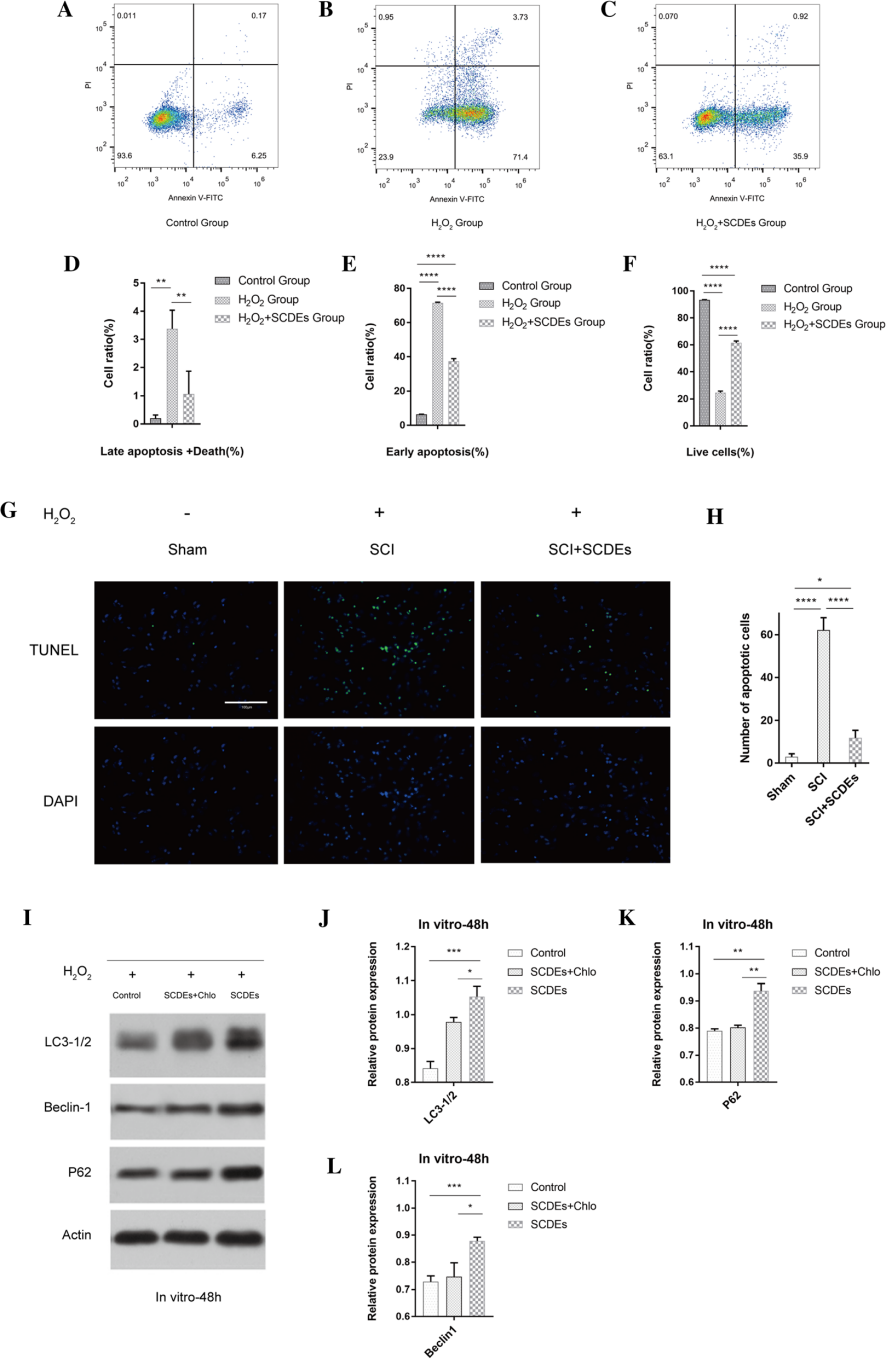
SCDEs inhibit apoptosis of injured neurons via autophagy in vitro. **A**–**C** Flow cytometry results demonstrated that H_2_O_2_-induced injury increased apoptosis in PC12 cells, which was reduced by SCDE treatment. Cells were classified as healthy cells (Annexin V − , PI −), early apoptotic cells (Annexin V + , PI −), late apoptotic cells (Annexin V + , PI +), and damaged cells (Annexin V − , PI +). **D**–**F** Quantification of late apoptosis, death, early apoptosis and live cells. **G** TUNEL staining identified that apoptosis was notably increased after H_2_O_2_-induced injury, whereas apoptosis was significantly decreased via SCDE treatment. **H** Quantification of the number of apoptotic cells. **I** Western blotting indicated that the SCDE-treatment group expressed higher protein levels of autophagic markers compared with those in other groups. **J**–**L** Semi-quantification of LC3-1/2, Beclin-1 and P62 expression. Scale bar, 100 μm. Data are presented as the mean ± SEM, two-way ANOVA with Tukey’s post-hoc test, n = 4. *P < 0.05, **P < 0.01, ***P < 0.001, ****P < 0.0001. *SCDEs* Schwann cell-derived exosomes

Next, H_2_O_2_-induced injury in PC12 cell-induced neurons was used to simulate SCI in vitro. The results of flow cytometry, used for assessing apoptosis, identified that H_2_O_2_-induced injury increased apoptosis in PC12 cell-induced neurons, which was reduced by SCDEs treatment (Fig. 3A–F). Furthermore, to confirm whether SCDEs regulated H_2_O_2_-induced injury in PC12 cell-induced neurons via autophagy, chloroquine was used to block autophagy. Chloroquine is frequently used clinically as an antimalarial agent, is a classic inhibitor of autophagy that blocks the binding of autophagosomes to lysosomes by altering the acidic environment of lysosomes, resulting in the accumulation of a large number of degraded proteins in cells (Golomb 1976; Mushtaque and Shahjahan 2015). TUNEL staining revealed increased neuronal survival in the SCDE treatment group (Fig. 3G and H). Western blotting results demonstrated that SCDEs increased the expression levels of LC3-1/2, Beclin-1 and P62 in PC12 cell-induced neurons subjected to H_2_O_2_-induced injury, whereas these SCDE-induced increases were decreased in the presence of chloroquine (Fig. 3I–L). These findings suggest that SCDEs inhibit the apoptosis of injured neurons via autophagy in vitro.

### SCDEs inhibit the activation of the Akt/mTOR signaling pathway via the downregulation of EGFR

**Fig. 4:**
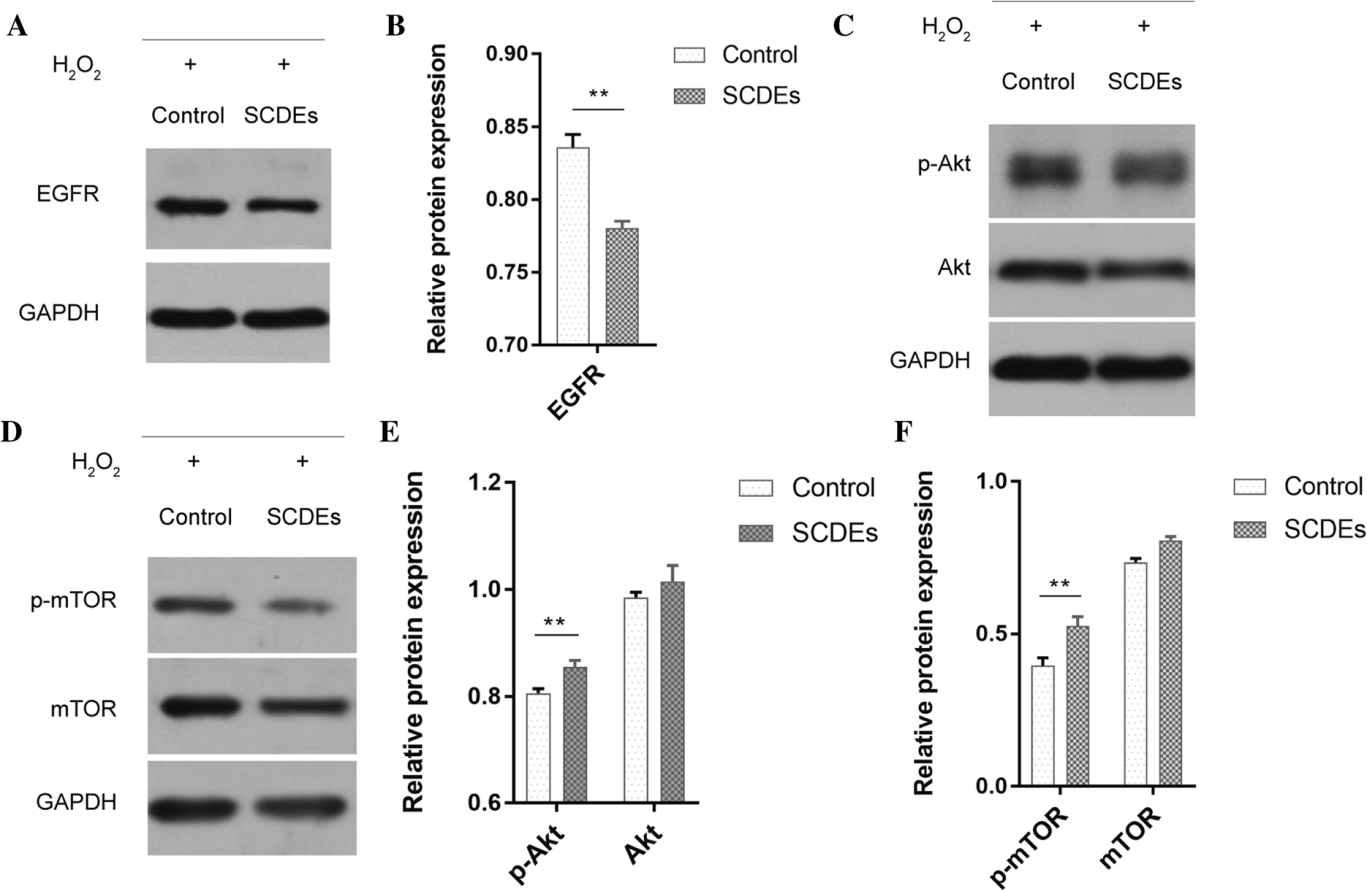
SCDEs inhibit the Akt/mTOR signaling pathway by downregulating EGFR. **A** Western blotting indicated that when H_2_O_2_-induced injury occurred, EGFR was downregulated after SCDE treatment. **B** Semi-quantification of EGFR expression. **C**, **D** The phosphorylation levels of p-Akt and p-mTOR were both notably decreased by SCDE treatment following H_2_O_2_-induced injury. **E**, **F** Semi-quantification of p-Akt, Akt, p-mTOR and mTOR expression. Data are presented as the mean ± SEM, two-way ANOVA with Tukey’s post-hoc test, n = 4. **P < 0.01. *SCDEs* Schwann cell-derived exosomes, *p-* phosphorylated

The western blotting results indicated that when H_2_O_2_-induced injury occurred, EGFR expression was downregulated after SCDEs treatment (P < 0.01; Fig. 4A and B). The Akt/mTOR signaling pathway, which was widely been considered to regulate autophagy(Heras-Sandoval et al., 2014; Zhang et al., 2020), localizes at the downstream target EGFR, and serves an essential role in regulating autophagic progression 34 (Dobashi et al. 2009; Fan et al. 2009; Li et al. 2016b; Ronellenfitsch et al. 2018; Treda et al. 2016). Therefore, western blotting was used to measure the phosphorylation levels of phosphorylated (p)-Akt and p-mTOR, both of which participate in autophagic regulation. Notably, SCDEs treatment decreased these phosphorylation levels in PC12 cell-induced neurons subjected to H_2_O_2_-induced injury (Fig. 4C–F). Collectively, the results suggested that SCDEs-induced autophagy in injured PC12 cell-induced neurons was associated with inhibition of the EGFR/Akt/mTOR signaling axis.

## Discussion

International Society for Extracellular Vesicles (ISEV) endorses “extracellular vesicle” (EV) as the generic term for particles naturally released from the cell that are delimited by a lipid bilayer and cannot replicate, i.e. do not contain a functional nucleus (Thery et al. 2018). SCI can instantly alter and compromise the function of multiple organs of the body, and often leads to irreversible damage and loss of sensory, motor and autonomic functions. The cost of care and the social burden associated with SCI are significant, as are the personal costs and injuries to the affected individuals (Ramer et al. 2014).

There are multiple treatments available for SCI, including surgical decompression, methylprednisolone, neuroprotection, magnesium, therapeutic hypothermia and cerebrospinal fluid drainage, as well as the most promising cell-based methods and bioengineering materials. However, the current efficacies of SCI treatments remain limited (Ahuja et al. 2017; de Rivero Vaccari et al. 2016).

The interaction between exosomes and recipient cells has been reported to enable exosomes to bind to cells via conventional receptor-ligand interactions, similar to those of cell–cell communication, membrane/fusion and endocytosis (Cordonnier et al. 2017; Simpson et al. 2008; Sun et al. 2018). It has been shown that exosomes carry biologically active cytokines, such as IL-1β, as well as an inflammasome component in the process of activating immune responses, such as via NLR family pyrin domain containing 3-induced inflammation (Qu et al. 2007). Additionally, exosomes regulate Toll-like receptor signaling and IL-1β production (Haneklaus et al. 2013). In promoting axonal regeneration after SCI, exosomes reduce PTEN activity by activating retinoic acid receptor β signaling, thereby reducing negative factors that inhibit axonal regeneration and promoting recovery after SCI (Goncalves et al. 2015).

Schwann cells are important components of axons in the peripheral nervous system. Studies have shown that Schwann cells secrete exosomes (Lopez-Verrilli and Court 2012), which promotes axonal regeneration both in vitro and in vivo (Ching et al. 2018). However, to the best of our knowledge, the influence of SCDEs on neurons has not been previously investigated. The present study demonstrated that SCDEs promoted the regeneration of axons, the recovery of motor function and the reduction of neuronal apoptosis following SCI.

Autophagy is one of the main pathways for cytosolic degradation and efficient conversion under stress (10). Autophagy involves three steps: Autophagosomal formation, autophagosomal and lysosomal fusion, and autophagic lysosomal degradation (Zhou et al., 2017). The most common mechanisms for upregulating the autophagic flux after traumatic brain injury include the following: increased levels of Beclin 1 protein and a decrease in the Beclin-1/BCL2 complex (Diskin et al. 2005; Kanno et al. 2011; Viscomi et al. 2012); enhanced type-III PI3 kinase activity; and autophagic processes. Additionally, it has been revealed that Beclin-1 proteins form a complex with *N*-methyl-d-aspartate receptor 2B (NR2B), which causes the dissociation of the Beclin-1/NR2B complex and the release of Beclin-1, which may contribute to induction of autophagy. Another potential mechanism may involve the gap junction protein, connexin 43, which directly interacts with the Beclin-1 complex and regulates autophagosomal biosynthesis, with some of the proposed pathways including mTOR, GSK3β and BCL2 interacting protein 3 (Bigford et al. 2009; Lin et al. 2014; Yu et al. 2013). The PI3K/AKT/mTOR pathway is claimed to regulate autophagy to induce apoptosis of alveolar epithelial cells in chronic obstructive pulmonary disease (Zhang et al. 2020) and modulate the clearance of protein to aggregate the neurodegeneration (Heras-Sandoval et al. 2014). Moreover, the PI3K pathway plays several crucial roles in neurogenesis by activating the proliferation, migration, and differentiation of neural stem cells (NSCs) (Koh and Lo 2015). And mTOR plays an important role in the regeneration of neurons, muscles, the liver, and the intestine, which are mainly mediated by mTORC1 rather than mTORC2 signaling (Wei et al. 2019). Previous studies have reported a close relationship between the different autophagy pathways and the biogenesis and secretion of exosomes. For example, one study revealed that the exosomal microRNA-7-5p mediated bystander autophagy by regulating the EGFR signaling pathway (Baixauli et al. 2014; Cordonnier et al. 2017; Li et al. 2016a; Song et al. 2016).

There were some limitations to the present study. For example, the present study did not upregulate or downregulate the expression levels of EGFR to verify the mechanism via which SCDEs repair SCI through this pathway. The current study also did not elucidate what specific constituents in SCDEs were involved in the regulation of EGFR and the inhibition of downstream pathways to putatively ameliorate SCI.

In conclusion, the present study is the first study to identify that SCDEs could induce axonal protection after SCI by increasing autophagy and decreasing apoptosis, and it was suggested that this may involve the EGFR/Akt/mTOR signaling pathway.

## Supplementary Information

Below is the link to the electronic supplementary material.

Supplementary file1 (DOCX 97 kb)

## Data Availability

The datasets used and/or analyzed during the present study are available from the corresponding author on reasonable request.
