# Supplementary material for: Autophagy induced by Schwann cell-derived exosomes promotes recovery after spinal cord injury in rats
Source: Biotechnol Lett. 2021 Nov 5;44(1):129–42. doi: 10.1007/s10529-021-03198-8 (PMC8854309; doi:10.1007/s10529-021-03198-8)
Supplement: Supplementary file 1 — Supplementary file1 (DOCX 97 kb) [file 10529_2021_3198_MOESM1_ESM.docx]

**Supplementary figure legends**


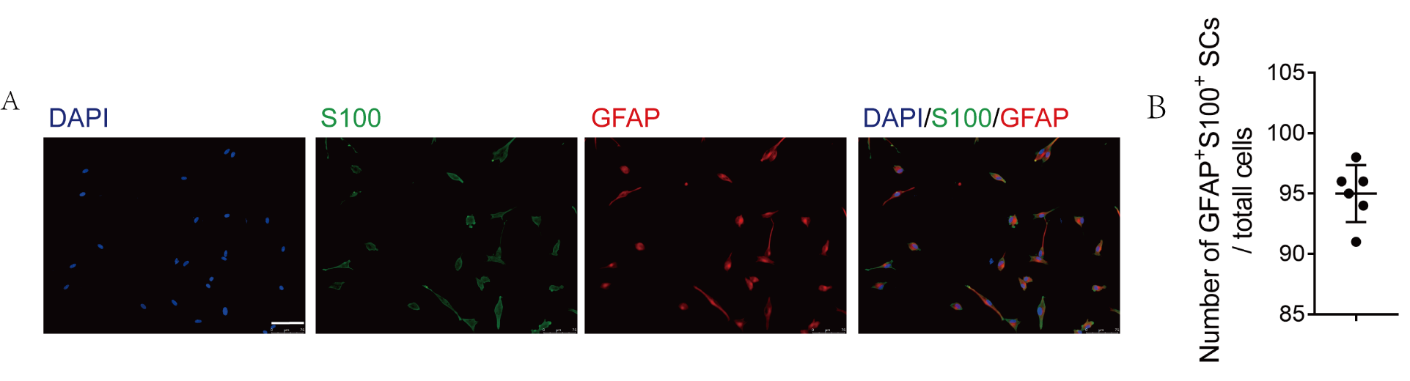


Figure S1. (A) Characterization of isolated Schwann cells. Immunofluorescent staining of S100 (green),GFAP (red) and DAPI. Scale bar, 75 μm. (B) Quantitative analysis of the number of GFAP+ and S100+ SCs/total cells.

Table 1 List of primary antibodies used in the present study.

| Antibody | Host | Company | Concentration |
| --- | --- | --- | --- |
| CD9 | Rabbit | Abcam | 1:2000 |
| CD63 | Rabbit | Santa Cruz | 1:200 |
| Alix | Rat | Cell Signaling Technology | 1:1000 |
| GAPDH | Rabbit | Cell Signaling Technology | 1:2000 |
| CHAT | Rabbit | Abcam | 1:1000 |
| NeuN | Mouse | Abcam | 1:200 |
| S100 | Rabbit | Abcam | 1:50 |
| GFAP | Chicken | Abcam | 1:500 |
